# Women in acute psychiatric units, their characteristics and needs: a review

**DOI:** 10.1192/pb.bp.115.051573

**Published:** 2016-10

**Authors:** Michaela Archer, Yasmine Lau, Faisil Sethi

**Affiliations:** 1 King's College London; 2 South London and Maudsley NHS Foundation Trust

## Abstract

**Aims and method** Recent policy guidelines published by the Department of Health highlight the need to develop gender-sensitive psychiatric services. However, very little is currently known about the specific characteristics and needs of female patients entering acute psychiatric wards, particularly psychiatric intensive care units. This article aims to review the current literature on what is known about this group of patients. PubMed, Embase and PsycINFO were systematically searched using a number of key terms.

**Results** A total of 27 articles were obtained. The findings were divided into four categories: admission characteristics, treatment needs, risk management and outcomes after discharge. Gender differences were found in diagnosis and presentation.

**Clinical implications** The differences observed in the reviewed studies suggest that women may have different assessment and treatment needs, and ultimately, different philosophies of care. A dearth of studies in this area indicates that if services are to develop in line with government policies, more research is needed.

Evidence suggests that mental health services have historically ignored the needs of female patients.^1^ In the 1990s, concerns regarding the lack of privacy and safety for women in psychiatric services led to calls for change.^2^ According to Bartlett & Hassell,^3^ these concerns were most evident in in-patient settings, where women were always in the minority, security levels were inappropriately high, and equality was not valued. These failings were investigated by the Department of Health,^4^ which led to the publication of policy guidelines on the provision of gender-sensitive care and women-only services.^5^ This was driven not only by safety concerns, but also by a growing body of evidence demonstrating gender differences within pathways into services, treatment needs, and treatment responses.^5^ These guidelines emphasised the development of services where women can feel safe, understood, and where staff are skilled in responding appropriately to issues such as violence, abuse, parenting roles, poverty and isolation.^6^

Studies have shown that single-gender wards are not necessarily safer or more gender-sensitive.^7^ The provision of a gender-sensitive approach requires staff attitudes to change and treatment programmes to be tailored to the needs of the women they are treating.^3^ However, research into what this should look like has been scarce, and there remains no clear, single approach to issues of gender, despite the government guidance.^8^ This is especially the case in secure and acute settings where the difficulties faced by female patients may be more subtle, and the hierarchical environment may feed into their feelings of powerlessness, exacerbating their illness.^3^ There is evidence that the needs of women in these services may have been particularly overlooked.^9^ This is worrying, given that women with a serious mental illness are considered to be more vulnerable to abuse and exploitation.^10^

Psychiatric intensive care units (PICUs) were developed to treat acutely disturbed patients whose behaviour could not be contained on general wards.^11^ A national audit of 33 adult PICUs (the majority were mixed-gender) found that they are not unique in the number of reports of sexual harassment and assaults against women.^12^ National minimum standards for PICUs^13,14^ have included such gender-related issues as the provision of gender-specific areas, ensuring equality of all treatments and assessment tools, ensuring self-care needs are met, and providing gender-sensitive groups. However, implementing these recommendations has presented practical issues, as the majority of PICU patients are male.^15^ Furthermore, the impact of these policies on building more gender-sensitive services has not yet been properly evaluated.^16^ Even more concerning is that there is no clear picture in the literature of what the specific needs of women admitted to PICU are. This paper will therefore review the existing literature on the characteristics and needs of women admitted to psychiatric in-patient services, with a particular focus on women admitted to secure units and PICUs.

## Method

PubMed, Embase and PsycINFO were systematically searched using the following key terms: ‘PICU’, ‘psychiatric intensive care unit’, ‘secure’, ‘ward’, ‘gender’, ‘gender-sensitive’, ‘difference*’, ‘women’, ‘female*’, ‘psychiatric’, ‘serious mental illness’, ‘acute mental disorder’. Using these terms, a manual search of the *Journal of Psychiatric Intensive Care* was also conducted to identify further relevant articles. Researchers from a feminist perspective have suggested that using a variety of research methods leads to a more holistic portrayal of the subject in question.^17^ In keeping with this perspective, the current review included the findings from both qualitative and quantitative studies. Specifically, studies that have focused on investigating gender differences in adult psychiatric in-patient services, in particular any studies that have investigated gender differences in PICU wards. This includes studies that have collected data from service users, staff and services in general. This was not limited to UK studies in order to obtain a more international picture of women's needs in these types of services. It is therefore acknowledged that, although PICU is a generally well-known term, in some countries the term emergency psychiatry may be used as a synonym for PICU.

## Results

**Fig. 1:**
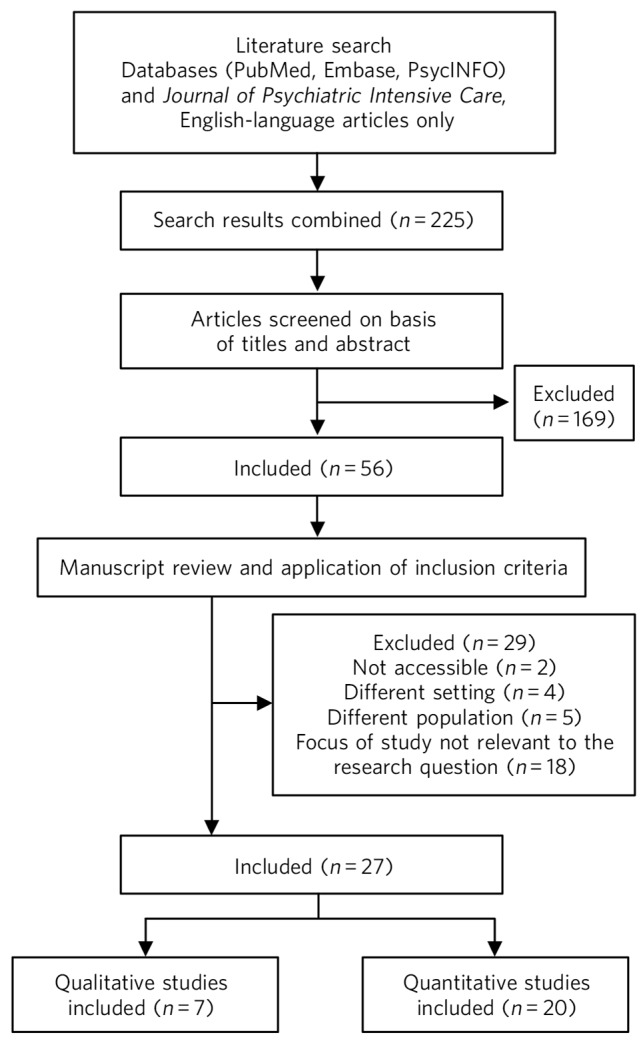
Study attrition diagram

The results of the database searches were manually screened and we removed articles that did not fit the selection criteria or had inappropriate content (Fig. 1). We found 7 articles that specifically related to PICU and 20 that concerned gender issues in general psychiatric in-patient settings. These were based in a variety of settings including low, medium and high secure units, general psychiatric hospitals, acute wards and forensic psychiatric wards. The relevant studies found in this review are listed in Table 1. The studies used both quantitative and qualitative methods and were not limited to the UK, although all were based in high-income Western countries. To understand the findings in more depth, the studies were divided into four categories relating to the different care pathways: admission characteristics, treatment needs, risk management and outcomes after discharge. This was so that the findings could be considered within the context of the relevant care pathway, as each has different implications for assessment and treatment. Within each category, findings from general and secure wards are discussed first, followed by the findings from articles specifically relating to PICU.

**Table 1:** Summary of results from literature search

Reference	Aims and design	*n*	Setting	Method and analysis	Main findings
Bowers *et al*^28^	Compared admissions and incidents across 3 units	Not reported	PICU	Retrospective multi-method analysis	Majority of admissions were young males, with psychosis
Bowers *et al*^25^	Literature review of the working model of PICU, patient profiles, etc.	N/A	PICU	N/A	Majority are male. No gender differences in satisfaction with care
Brown & Bass26	Compared PICU and non-PICU patients in same hospital	330 (114 female)	PICU	Retrospective case-note analysis	Patients predominantly male. Females may be particularly disturbed, higher rates of violence to self and others, personality disorder diagnosis more common
Brown *et al*^27^	Describes admissions and outcomes from 7 wards	332 (72 female)	PICU	Prospective, multi-centre case analysis	Majority male. More females in relationships, fewer misused drugs/alcohol, self-harm more common, more diagnosed with personality disorder
Gintalaite *et al*^30^	Describes characteristics and outcome of first female-only unit	49 female patients	PICU	Prospective case-note analysis	Majority single; with psychosis or personality disorder; transferred for aggression and self-harm. Average length of stay 8 weeks, but longer with personality disorder
Gramaglia *et al*^37^	Assessed gender differences in sample of first admission in patients with and without substance use disorder	1473 (819 female)	Psychiatric ward, Italy	Retrospective case-note analysis	Divorce, family problems and self-harm were all found to be risk factors for comorbidity in females
Hietanen & Punamaki^35^	Studied link between attachment styles and working alliance	100 in-patients (62% female), 21 staff	Acute unit, Finland	Data collected from self-report questionnaires	Adult attachment style associated with working alliance, but association was different in men and women
O'Brien *et al*^15^	Gender differences in admission, incidents and outcomes	91 (14 female)	PICU	Retrospective case-note analysis	Fewer women referred and tended to stay longer
Mustafa *et al*^29^	Gender differences in admission and referral pathways	206 (64 female)	PICU	Retrospective case-note analysis	Males more likely admitted for aggression and had higher substance misuse
Beer *et al*^42^	Measured treatment effectiveness and predictors of change	86 (25 female)	Low secure, UK	Case series	Improvements found on HoNOS. Female gender associated with deterioration on scores
Berg^22^	Studied diagnostic differences in referrals	998 (480 female)	Acute unit, Norway	Retrospective case series	Women with personality disorder more common, men with substance misuse more common
Coid *et al*^18^	Gender differences in admission characteristics	3005 (450 female)	High/medium secure, UK	Retrospective case-note analysis	Gender differences found in diagnosis, comorbidities and forensic history
Coid *et al*^43^	Measured re-offending following discharge	1344 (177 female)	Medium secure/ community, UK	Follow-up study	Risk of re-conviction higher in men
Cutting & Henderson^33^	Examined women&s experience of in-patient psychiatric care	32 female patients	Psychiatric hospital, UK	Focus groups and interviews	Dissatisfied with many aspects of care including mixed-gender wards
Dickens *et al*^39^	Compared incident data across care pathways	N/A	Medium/low secure, UK	Retrospective survey	Women more likely to be involved in other-directed and self-harm incidents
Krakowski & Czobor^38^	Examined gender differences in violent behaviours	189 (67 female)	Psychiatric hospital, USA	Prospective analysis of incidents	Gender differences in patterns of violent behaviours and impact of certain risk factors
Lart *et al*^24^	Literature review of women in secure psychiatric services	N/A	N/A	Systematic review	Women had a wide range of needs and were different to men in significant ways
Long *et al*^20^	Describes development of best-practice treatment for women	27 female patients	Medium secure, UK	Descriptive case study	Majority have comorbid personality disorder, experiences of abuse, and offences of manslaughter/arson
Long *et al*^32^	Explored service users' views on effective therapeutic milieu	19 female patients	Low/Medium Secure, UK	Service user-led focus groups	Themes identified: interpersonal relationships, treatment programming, service user empowerment, safety and hope for future
Long *et al*^21^	Mapped the characteristics of women over a 6-year period	65 female patients	Medium secure, UK	Prospective case-note analysis	Most had primary diagnosis of personality disorder, plus histories of violence and self-harm
Maatta^31^	Explored patients' experiences of psychiatric care	N/A	Psychiatric hospital, UK	Literature review	Main themes were women want a broad range of treatments, and prefer single-gender units
Maden *et al*^44^	Investigated gender differences in re-offending over 1 year	959 (116 female)	Medium secure, UK	Follow-up study	Women less likely to be reconvicted. Adjustments for self-harm, drug/alcohol problems and previous offending reduced differences
Nathan *et al*^41^	Compared risk of burnout in nurses	28 nurses	Medium secure, UK	Level of burnout assessed before and after ward opened	Nurses working on women's ward experienced more emotional exhaustion
Nicholls *et al*^23^	Examined the risk profiles of female patients and contrasted with male counterparts	527 (12% female)	Secure forensic unit, Canada	Retrospective file reviews	Women no less likely than men to have a violent index offence and to perpetrate in-patient aggression
Sahota *et* *al*^19^	Investigated gender differences in characteristics and outcomes	595 (93 female)	Medium secure, UK	Retrospective follow-up data analysed	Differences found in admission characteristics and outcome
Schon^34^	Explored service user views on compulsory in-patient care	30 (15 female)	Psychiatric hospital, Sweden	Service user interviews	Stories of coercion more common in women. Women emphasised need for emotional support
Somers & Bartlett^40^	Explored the nature and quality of care pathways for women	47 local experts	Low/medium secure, UK	Psychiatrists and ward managers interviewed	Focused on physical relocation. Care promoted by increased awareness of women's needs, continuity of care and support for teams

### Admission characteristics

Within secure in-patient settings, men are consistently more likely to be admitted than women.^18^ Women who are admitted tend to have fewer previous convictions, more previous psychiatric admissions, and are more likely to be admitted from hospital than prison.^18,19^ Women with a forensic history are significantly more likely to have been charged with or convicted of arson.^18,20,21^ Female patients are more likely to have a diagnosis of major depressive disorder and personality disorder, borderline personality disorder in particular,^18,19,21–24^ whereas male patients are more likely to have a diagnosis of schizophrenia, and to have comorbid substance misuse.^19,22^ Women are also more likely to present with a range of other conditions, such as eating disorders and anxiety disorders.^18^ In terms of their histories, they are more likely to present with a history of self-harm and physical and/or sexual abuse.^19,20^ A review of women in secure services concluded that they represent a diverse group with individual personal, psychiatric and forensic histories.^24^ However, the current review revealed some trends that have implications for assessment and treatment, such as that women are more likely to be diagnosed with a personality disorder, and to have histories of physical and/or sexual abuse.

Female patients admitted to PICU are also consistently in the minority.^25^ Most studies in this area have not included gender in the analysis, which means that the findings mainly describe male PICU patients. These studies show that the majority of PICU patients are typically male, White, in their thirties, and have a primary diagnosis of schizophrenia.^15,25–28^ Patients often have histories of substance misuse, violence and aggression.^26,27,29^ Where studies have included gender in the analysis, there is the problem of small sample sizes and thus a lack of power. Brown *et al*'s^27^ study had a relatively large cohort (*n* = 332), but of these only 72 (21.5%) were female. Another study^29^ had a smaller sample size (*n* = 206), but assessed a higher percentage of female in-patients (*n* = 64, 31%). We can tentatively suggest that women admitted to a PICU may be more likely to be in a relationship, self-harm is more common but no more severe than in men, and fewer women than men misuse drugs or alcohol.^27,29^ No gender differences have been found in symptom severity.^26^ There was disagreement about whether there are gender differences in primary diagnosis within the three studies found that investigated this. Two studies suggested that female PICU patients may be less likely to have a primary diagnosis of schizophrenia, and more likely to be diagnosed with personality disorder.^26,27^ The other study found no differences.^29^ One study that looked exclusively at the characteristics of female PICU patients found that the majority were White, and had diagnoses of either schizophrenia or personality disorder;^30^ 77% of the total sample (*n* = 49) had histories of physical aggression and severe self-harm, and had a number of previous admissions to mental health wards. Another study suggested that female patients may have significantly higher rates of violence and self-harm before their PICU admission than male PICU patients.^26^ However, this study had a small sample size (*n* = 165) and only 37 patients (22.5%) were female. It also used a retrospective design and data were taken from only one unit, which significantly limits the generalisability of these findings. Overall, these studies suggest that women are as unwell as men on admission to a PICU, but there may be differences in diagnosis and presentation.

### Treatment needs

We found a limited number of studies that directly compared the treatment needs of men and women. However, a number of qualitative studies have conducted in-depth explorations of what women want from services. A systematic review^31^ of male and female experiences in a psychiatric ward found only five relevant articles. These findings suggested that women prefer to have a broad range of treatment options, including therapy groups, complementary therapy and writing activities. Women also preferred being in a single-gender environment, as they felt safer and more able to talk to other women about their experiences. Long *et al*^32^ conducted a focus group with women (*n* = 19) who were volunteers from medium and low secure units. Most of the patients had a dual diagnosis of personality disorder (mainly borderline personality disorder) and mental illness. Thematic analysis revealed five factors to which the patients attached greatest importance: the development of good interpersonal relationships with staff, who are understanding, empathetic, have insight, and get to know the patients beyond their mental illness; well-planned and meaningful activities; acknowledgment of progress; having clear rules; and a dual emphasis on physical and mental needs.^32^ Other important factors included an individualised approach that promotes empowerment, a safe and settled environment with emphasis on de-escalation, and the importance of instilling hope by having future-oriented goals.^32^ These findings have been supported by qualitative studies of women's experiences in general in-patient psychiatric services.^33,34^ One study that compared the experiences of men and women recovering from a severe mental illness found that women are more likely to emphasise the need for emotional support and trusting relationships with staff.^34^ Another study found that women on an acute in-patient ward with an insecure attachment style had more difficulties forming emotional and relational bonds than securely attached men and women, as well as insecurely attached men.^35^ This suggests that having an understanding of attachment theory^36^ and how this may influence the therapeutic alliance may be important when working with women. A study comparing male and female in-patients with comorbid substance use disorder found that having family problems and being divorced was a risk factor for women but not for men.^37^ The authors suggested that a treatment approach focusing on distress, family problems and relational issues may be more beneficial for women.

No studies were found that explored the treatment needs of women, specifically within PICU. A literature review of the working model of PICU suggests that patients generally prefer structure, routine and a good number of activities to keep them occupied.^25^ Staff interactions with patients were also noted as important aspects of care in PICU, including enabling the patient to feel heard, understood and treated with respect. O'Brien *et al*^15^ found that women tended to receive longer-term care than men. However, this study is limited by a small sample size (*n* = 91), and is based in only one London trust. Gintalaite-Bieliauskiene *et al*^30^ found that some patients with personality disorder had excessively long in-patient stays in PICUs. Research needs to be carried out to investigate the needs of women in PICU to address this gap in the literature. It may also be necessary to investigate the question of why some patients with a personality disorder may be staying in hospital longer than is appropriate, as PICUs are not designed for long-term care.

### Risk management

A number of quantitative studies have analysed incidents of violence and aggression on in-patient wards. It is generally found that female in-patients are aggressive at similar rates and severity levels as male in-patients, although they may be less likely to cause injury to others.^23^ Krakowski & Czobor^38^ found that women had a much higher level of verbal assaults and early physical assaults. Difficulties with ward routine and social interactions were found to be more highly associated with aggression in these women. A more recent survey of incidents on medium and low secure units^39^ found that women were more likely to be involved in other-directed and self-harm incidents, but that men were involved in the most severe incidents. The higher level of intensive observations required for women who self-harm, as well as the smaller number of women in services, means that caring for women is generally considered more resource intensive than for men.^40^ This may contribute to the feeling often reported by staff that female patients are more demanding to work with, and may help explain the higher level of burnout and emotional exhaustion associated with working on a female-only ward.^41^

The literature on gender differences in risk management in PICUs is extremely scarce. One study has suggested that, compared with men, women in PICUs present with significantly higher rates of violence towards themselves and others during their admission; they may also be more likely to be placed in seclusion and to be prescribed antidepressants and mood stabilising drugs.^26^ However, this study is limited as it had a relatively small sample size (*n* = 165), uses a retrospective design, and is based on only one unit.

### Outcomes after discharge from in-patient services

Gender differences have been found in outcomes at the point of discharge for women in psychiatric in-patient settings. A study analysing Health of the Nation Outcome Scales (HoNOS) found that on discharge, deterioration in scores was associated with female gender.^42^ This implies that women may not show the same improvements that men do. Another study found that women have a significantly higher chance of being readmitted to a psychiatric hospital than men.^19^ In contrast, the risk of reconviction for committing a criminal offence is higher for men.^19,43^ However, once history of self-harm, substance misuse and previous criminal histories were adjusted for, the gender differences in reconviction rates were reduced.^44^ There are no studies directly relating to outcomes for female patients discharged from PICU.

## Discussion

This review has identified a number of characteristics that women in in-patient psychiatric settings tend to present with. First, the evidence presented here suggests that the need for good interpersonal relationships with staff is a central aspect of caring for women admitted to psychiatric wards. Numerous qualitative studies have highlighted this issue,^32–34^ and emphasise that staff should be understanding, empathetic, insightful, and should get to know the patient beyond their illness. This may be linked to the finding that there is a higher co-occurrence of personality disorders and histories of abuse or trauma in female in-patients. Therefore, developing a good therapeutic alliance could be a crucial part of treatment for patients who are more likely to be sensitive to feelings of abandonment and rejection.^40^ Previous research has suggested that women are more socially oriented than men.^45^ However, social interactions were found to be more highly associated with aggression in females than in males.^38^ Therefore, in order to maintain a therapeutic environment, good interpersonal relationships with staff, who also have an awareness of attachment difficulties, may be more valuable for women in the acute phases of mental illness. This has been supported by studies showing that positive perceptions of the ward environment were associated with higher levels of therapeutic alliance.^46^

Second, this review suggests that a clear routine and structure within the ward, as well as a full activity programme, is important in order to maintain a safe and settled environment. This was supported in a number of qualitative studies focusing on the experiences of women in secure settings. This may also be equally important for men in secure settings. However, evidence has suggested that there is a stronger link between female aggression and ward routine.^38^ Therefore, having a clear routine may be especially important for women in helping to reduce violence and aggression rates. This may also link to the increased prevalence of personality disorder in female in-patients, as studies have suggested that a structured, goal-oriented approach, with consistent treatment boundaries, is effective for treating personality disorder.^47^

Third, this review suggests that women tend to present with higher rates of comorbidity with other mental health problems (including personality disorders, eating disorders and anxiety disorders), as well as a higher occurrence of trauma, abuse and self-harm. Evidence suggests that these factors are all significant predictors of poor outcomes in longitudinal studies.^48^ The increased prevalence of personality disorder and the fact that women are more likely to have been victims of violence in the past have also been linked to higher rates of aggression in female patients.^49^ This complex constellation of problems may contribute to the finding that women tend to have longer lengths of stay at PICUs;^15,30,50^ and may also be linked to the higher levels of burnout reportedly experienced by staff working on female wards.^41^ Previous researchers have made recommendations for increased relational security due to the higher level of risk (to staff, other patients and themselves) than that found in men.^24,51^ Relational security emphasises more individualised levels of security, skilled nursing care and psychological therapies. This highlights the importance of services providing a high level of staff support and training so that they are able to safely contain and manage these complex problems while also providing access to appropriate therapeutic interventions.^52^ Researchers have found that providing staff with gender-informed training can greatly improve the experience and recovery process for patients.^53^ Therefore, the findings of this review also highlight the need for staff training, supervision and competence in dealing with these complex issues sensitively.

It has been argued that, despite 20 years of research in this area, evidence that there is a clear understanding of what works for this group remains scant.^54^ This was found to be the case in this review, especially in terms of the number of articles available on gender differences in PICU. Although it is possible to make some suggestions and recommendations about women in secure or general in-patient wards based on the current evidence base, this is not possible about women in PICUs. This suggests that there is a lack of awareness about the characteristics and needs of this group. For example, one of the main findings from the present review was regarding the gender differences in diagnosis and the higher prevalence of personality disorder in women in secure and general in-patient wards. However, there is no strong evidence to support this difference in PICU due to the lack of robust studies. In the past, research findings from men in PICU have been generalised to women, and treatment or care pathways were applied to women in the same way as they were to men. It has also been argued that mental health services have often failed women by ignoring the sources of their distress, medicalising what are in fact social difficulties, and failing to recognise their roles as mothers.^55,56^ PICUs have struggled to address these issues, and there is evidence that this has been due to practical problems.^15^ However, there is also the possibility that the higher emphasis placed in PICU on medical and behavioural management of symptoms means that a focus on the specific needs of women can be easily lost.

Services have made progress since then, and new national minimum standards for PICUs and low secure units^57^ have now been published. These standards recognise that women do have different needs to men in a number of areas including the physical environment, risk management, assessment and staffing. However, there remains a need for the characteristics of this population to be mapped out clearly, so that treatment can be adapted to meet their needs.^21^ Developing services that are tailored to meet the needs of men and women, and evaluating the impact of these treatment programmes, are seen as a research priority by the government.^5^ The continuation of research in this area is therefore crucial if services are to develop gender-sensitive treatment programmes.

## Strengths and limitations

This review provides a fresh overview of a topic that may have been overlooked until now. The findings can be utilised by services interested in developing a more gender-sensitive approach. However, the findings also highlight the fact that this group of women is not homogenous.^24^ There may also be many similarities between male and female patients; what is important for female in-patients may also be important for the care of male in-patients. However, this article is part of a wider research project aimed at addressing this issue by gathering more evidence on what factors may be more important for women in PICUs compared with men.

The main limitation of this review is that the ability to draw any firm conclusions based on the current academic literature, particularly regarding PICU, is extremely limited. Most of the articles found focus on admission profiles of women, but are limited by small sample sizes. This indicates a pressing need for more robust studies to be carried out on admission characteristics, and for further research to be conducted on outcomes and effective treatments. For example, analysing gender differences in risk profiles and outcomes within PICU, or interviewing staff and service users to gather their views on what women requiring intensive psychiatric care need. Finally, this review did not include searches for relevant policy reports, which present a resource that could be utilised in future reviews of the needs of female in-patients.
